# Challenges, issues and trends in fall detection systems

**DOI:** 10.1186/1475-925X-12-66

**Published:** 2013-07-06

**Authors:** Raul Igual, Carlos Medrano, Inmaculada Plaza

**Affiliations:** 1 R&D&I EduQTech Group, Escuela Universitaria Politecnica de Teruel, University of Zaragosa, Teruel, Spain

**Keywords:** Fall detection, Review, Smart phones, Assistive technology, Health care

## Abstract

Since falls are a major public health problem among older people, the number of systems aimed at detecting them has increased dramatically over recent years. This work presents an extensive literature review of fall detection systems, including comparisons among various kinds of studies. It aims to serve as a reference for both clinicians and biomedical engineers planning or conducting field investigations. Challenges, issues and trends in fall detection have been identified after the reviewing work. The number of studies using context-aware techniques is still increasing but there is a new trend towards the integration of fall detection into smartphones as well as the use of machine learning methods in the detection algorithm. We have also identified challenges regarding performance under real-life conditions, usability, and user acceptance as well as issues related to power consumption, real-time operations, sensing limitations, privacy and record of real-life falls.

## Introduction

According to the World Health Organization [1] approximately 28-35% of people aged 65 and over fall each year increasing to 32-42% for those over 70 years of age. The frequency of falls increases with age and frailty level. In fact, falls exponentially increase with age-related biological changes, which is leading to a high incidence of falls and fall related injuries in the ageing societies. If preventive measures are not taken in the immediate future, the number of injuries caused by falls is projected to be a 100% higher in 2030. In this context, assistive devices that could help to alleviate this major health problem are a social necessity. Indeed, fall detectors are being actively investigated.

A fall detection system can be defined as an assistive device whose main objective is to alert when a fall event has occurred. In a real-life scenario, they have the potential to mitigate some of the adverse consequences of a fall. Specifically, fall detectors can have a direct impact on the reduction in the fear of falling and the rapid provision of assistance after a fall. In fact, falls and fear of falling depend on each other: an individual who falls may subsequently develop fear of falling and, viceversa, the fear of falling may increase the risk of suffering from a fall [2]. Fear of falling has been shown to be associated with negative consequences such as avoidance of activities, less physical activity, falling, depression, decreased social contact and lower quality of life [3]. The effect of automatic fall detection units on the fear of falling has been studied by Brownsel et al. [4]. They conducted a study with community alarm users who had experienced a fall in the previous six months. At the end of the study, those who wore the fall detector appropriately reported that they felt more confident and independent, and considered that the detector improved their safety. One of the conclusions of the study was that the fear of falling is likely to be substantially affected by user perception of the reliability and accuracy of the fall detector.

The other important aspect that fall detectors may help to reduce is the time the elderly remain lying on the floor after falling (long lie). This time is one of the key factors that determine the severity of a fall. Many older fallers are unable to get up again without assistance and any subsequent long lie can lead to hypothermia, dehydration, bronchopneumonia and pressure sores [5,6]. This is particularly critical if the person lives alone or loses consciousness after falling. Lord et al. [7] reviewed different studies on the long lie. They found that the long lie is a marker of weakness, illness and social isolation and is associated with high mortality rates among the elderly. More than 20% of patients admitted to hospital because of a fall had been on the ground for an hour or more, and even if there was no direct injury from the fall, their morbidity rates within 6 months were very high. Robust fall detectors may have the potential to diminish this long lie. A robust fall detector is one that is able to classify the falls as falls and the non-falls as non-falls even under real life conditions. If a fall event occurs and the system does not detect it, the consequences can be dramatic: the person can remain lying on the floor for a long time with all that this implies. In addition, the loss of confidence in the system may eliminate the benefits of the detector on the fear of falling. By contrast, if the system reports an excessive number of false activations, caregivers may perceive it as ineffective and useless, which may lead to device rejection. But robustness is not easy to achieve. Although several commercial products are available on the market, the fact is that they are not widely used and do not have a real impact on the elders’ lives yet [8,9]. Besides, the vast majority of their potential users do not know of their existence. However, when the concept of fall detection is presented, they find in it a great potential to improve their security and safety in home.

For these and many more reasons, the number of studies on fall detection has increased dramatically over recent years. Unfortunately, there are not many reviews on fall detection. The work of Noury et al. [10], which appeared in 2008, can be considered the first one in this field. Shortly thereafter, Perry et al. [11] published a similar analysis. These studies provided a general overview of the fall detection status, but it has changed greatly since they were published, and the current fall detection trends have little in common with those of previous years. Mubashir et al. [12] is more recent, but it includes only 2 papers from 2011 and lacks later papers anyway, for instance many smartphone-based detectors. In our study, we do not present a detailed discussion of each paper, like [12], but rather we show the information schematically by means of tables including relevant information for people conducting research in this field: the types of falls used in the simulations, the number of users involved in the tests, whether they include data from older people, detectors’ performance, methods and features used for classification, etc. In addition we have performed a longitudinal study to identify the current trends. We have also included our view of the challenges that fall detection faces and we have highlighted the critical issues that can compromise its use in real-world scenarios. We hope that our work will serve as a reference for both clinicians and biomedical engineers planning or conducting field investigations.

The study has been carried out through the analysis of several journal articles and conference proceedings. A search of IEEE Xplore, PubMed, MEDLINE, Google Scholar, and Web of Knowledge has been conducted. The search strategy included either “fall detection”, “fall detector”, “detection of falls”, “automatic fall detection”, “fall detection mobile phones”, “fall detection accelerometers”, “fall detection context” or “fall events” as the keywords. References were searched by hand and further relevant papers identified from their citations. A total of 327 studies on fall detection were found, which were then categorized into different groups (see section *Classification of fall detection systems*). Some of them were selected for a further analysis that is presented in this paper. The selection criteria were:

–Firstly, we considered the most cited study per year in each category, from 2005 to 2012, according to the ranking provided by the Web of Knowledge.

–Secondly, among the remaining studies, we made a personal selection in order to obtain those works that may help to identify the challenges, issues and trends in fall detection as well as to provide a comprehensive vision of the different detection techniques and the current status of this field. Only studies including some experimental results or pioneering investigations have been considered.

The rest of this paper is organized as follows: firstly, a classification of fall detection systems is presented, distinguishing between context-aware systems and wearable devices; secondly, we discuss the challenges, issues and trends in fall detection.

### Classification of fall detection systems

The literature reviewed provides evidence of the lack of a common approach. Noury et al. [10] classify the different studies on fall detection according to whether they only focus on the detection of the impact shock, or they also include the detection of the postfall phase. By contrast, Mubashir et al. [12] divide fall detectors into three categories: wearable device based, ambience sensor based and camera (vision) based. Perry et al. [11] group them into three categories: methods that measure acceleration, methods that measure acceleration combined with other methods, and methods that do not measure acceleration.

Essentially, the structure of all fall detection systems is always similar. Their main objective is to discriminate between fall events and activities of daily living (ADL). This is not an easy task as certain ADL, like sitting down or going from standing position to lying down, have strong similarities to falls. Thus, in order to test a fall detector, it is necessary to collect data from falls and ADL, which can be real (very difficult, especially for falls) or simulated by young volunteers (a feasible option adopted by most authors). These data are recorded by sensors and can be in form of acceleration signals, images, pressure signals, etc. Then, they are processed and classified using a fall detection technique capable of distinguishing between falls and ADL. In most cases, the performance of the detector is expressed in terms of sensitivity (SE) and specificity (SP). The sensitivity is the ability of a detector to correctly classify a fall as a fall, while the specificity is the ability of a detector to correctly classify an ADL as ADL [13].

After reviewing the literature, we conclude that fall detectors can be broadly categorized into two types: context-aware systems and wearable devices. The first category includes 151 studies, while 197 papers belong to the second one. Twenty-one studies have been included in both categories since they use a combination of techniques. Next sections investigate some of the most relevant studies in both groups.

### Context-aware systems

These systems use sensors deployed in the environment to detect falls. Their main advantage is that the person does not need to wear any special device. However, their operation is limited to those places where the sensors have been previously deployed [14].

Among all the possible types of sensors, the most common are cameras, floor sensors, infrared sensors, microphones and pressure sensors. Video-based systems can be considered as a subcategory in this group as they use computer vision techniques that differ from the rest of the detection methods. Table 1 compares some of the most significant works in this area. For the comparison, we suggest a set of 9 items: the year of their publication, a brief description of the fall detection technique, the features extracted to perform the fall detection, the fall types considered in the study, the subjects involved in the testing phase, the type of sensor used and whether they include data from older people or not.

**Table 1:** Comparison of context-aware systems

**Article**	**Year**	**Basis**	**Features used for fall detection**	**Fall types**	**Subjects**	**Declared performance**	**Type of sensor**	**Elders *Yes/No***	**Comments**
Lee *et al.*[20]	2005	Vision-based method for monitoring falls at home	State and geometrical orientation of the silhouette at time t, spatial orientation and speed of the centre of the silhouette	Fall lying down in a ‘stretched’ position and fall lying down in a ‘tucked’ position	21 subjects (age 20–40)	SP: 80.5%	Camera	No	Personalized thresholds are established based on the height of the subjects
						SE: 93.9%			
Miaou *et al*. [15]	2006	Customized fall detection system using omni-camera images	The ratio of people’s height and weight	Not specified	20 subjects	With personal information: SP: 86% SE: 90%	Camera	No	Determining a proper threshold statistically for different ranges of height or weight alone does not improve the system performance
Vishwakarma *et al.*[21]	2007	Automatic detection of human fall in video	Aspect ratio, horizontal and vertical gradient distribution of object in XY plane and fall angle	Sideways, forward, backward falls	1 subject	SP: 100% SE: 100%	Camera	No	Both indoor and outdoor video containing different types of possible falls are taken
Cucchiara *et al.*[19]	2007	A multi-camera vision system for detecting and tracking people and recognizing dangerous behaviours	Geometrical and colour features together with the projection of the silhouette’s shape on the x and y axes.	Not specified	Not specified	Difficulties with occlusions are reported	Camera	No	If a fall is suspected it delivers live video streams to clinicians in order to check the validity of a received alarm
Fu *et al.*[16]	2008	Contrast vision system designed to detect accidental falls	Change in illumination	Backward, forward and sideways falls	3 subjects	3 possible scenarios evaluated with positive results	Contrast vision sensor	No	Instantaneous motion vectors are computed and fall hazards are immediately reported with low computational effort
Hazelhoff *et al.*[17]	2008	Real-time vision system to detect fall incidents in unobserved home situations	The orientation of the main axis of the person and the ratio of the variances in horizontal and vertical direction Skin colour	Not specified	At least 2 subjects	SE: 100% when large occlusions are absent	Camera	No	The position of the head is taken into account in order to obtain a high robustness
Anderson *et al.*[22]	2009	3D representation of humans (voxels) using multiple cameras. Two levels of fuzzy logic determines first a state and then activities (f.i. a fall)	At low level: silhouettes from each camera, to build a set of voxels. At an intermediate level: centroid, height, major orientation of the body and similarity of the major orientation with the ground plane normal.	At least, falls forward, backwards, and to the side (with recovery, attempting to get back up, lying motionless)	Not specified	SE: 100%	Camera	No	The system can produce sentences like “the person is on-the-ground in the kitchen for a moderate amount of time”
						SP: 93.75%			
Lie *et al.*[23]	2010	Vision fall detection system considering privacy issues	The ratio and difference of human body silhouette bounding box height and width	Not specified	15 subjects (age 24–60)	Accuracy 84.44%	Camera	No	Activities are divided into three categories: standing posture, temporary posture and lying down posture
Rimminen *et al*. [28]	2010	Fall-detection method using a floor sensor based on near-field imaging	Features related to the near-field imaging floor (the number of observations, the sum of magnitudes and dimensional features)	Backward to sit, backward to lateral, to supine, onto knees, arm protect, to prone, rotate right and left, right and left lateral	10 subjects	SE: 91%	Near-field image sensor	No	The fall-detection performance is valid for multiple people in the same room
						SP: 91%			
Tzeng *et al.*[25]	2010	A system that adjusts the detection sensitivity on a case-by-case basis to reduce unnecessary alarms	Floor pressure signal	Backward, forward and sideways falls	Not specified	SP: 96.7%	Pressure/ infrared sensors	No	The floor pressure sensor is combined with the infrared sensor
			Image features: standard deviation of vertical projection histogram, standard deviation of horizontal projection histogram, and aspect ratio			SE: 100%			
Diraco *et al.*[24]	2010	An active vision system for the detection of falls and the recognition of postures for elderly homecare applications.	People’s silhouette and their centre-of-mass	Backward falls, forward falls, lateral falls	Not specified	SE: 80%	Camera	No	Information about the 3D position of the subject is combined with the detection of inactivity.
						SP: 97.3%			
									An approach for posture recognition is proposed
Rougier *et al.*[14]	2011	A vision system based on analyzing human shape deformation	Some edge points from the silhouette of the person	Forward falls, backward falls, falls when inappropriately sitting down, loss of balance	Not specified	Accuracy (falls and ADL correctly classified): 98%	Camera	No	The fall impact is an important feature to detect a fall, but the lack of movement after the fall is crucial for robustness
Li *et al.*[29]	2012	Acoustic fall detection system	Acoustic signals sampled at 20 KHz	Backward, forward and sideways falls (balance, lose consciousness, trip, slip, reach chair, couch)	3 subjects (2 female, 1 male, ages 30, 32, 46)	SE: 100%	Array of microphones	No	The source of the sound is located.
						SP: 97%			
									The performance of the acoustic detector is evaluated using simulated fall and nonfall sounds
Mastorakis *et al.*[18]	2012	Real-time fall detection system based on the Kinect sensor	The width, height and depth of the human posture, which define a 3D bounding box	Backward, forward and sideways falls	8 subjects	All falls were accurately detected	Infrared sensor	No	The system requires no pre-knowledge of the scene and three parameters to operate; the width, height and depth of the subject
Zhang *et al.*[27]	2012	Privacy Preserving Automatic Fall Detection	Deformation and person’s height	Fall from chair, fall from standing	5 subjects	Accuracy 94%	RGBD cameras	No	The system can handle special cases such as light turning off (insufficient illumination)

There is a high variability in detection techniques as they are dependent on the type of sensor used. All methods start with a feature extraction, for example, the ratio of people’s height and weight [15], the edge points from the silhouette of a person [14], changes in illumination [16], the orientation of the main axis of the person [17], the width, height and depth of the human posture [18], the skin colour to detect people [19], etc. Then these features are compared and classified to distinguish normal activities from real falls using different techniques (Table 2). At present, lots of different features have been examined and none of them prevails over the rest since they give similar results and no comparison has been done.

**Table 2:** Fall detection techniques in the context-aware studies

	**Fall detection method**				
	**1**^**st **^**step**	**2**^**nd **^**step**	**3**^**rd **^**step**	**4**^**th **^**step**	**5**^**th **^**step**
Lee *et al.*[20]	Adaptive background subtraction to detect the object of interest	Image processing using a connective-component labelling technique, with the end product being a ‘blob’ or silhouette	Feature extraction	Determination of the threshold values for each of the features based on the height of the users	
Miaou *et al.*[15]	Background subtraction to detect the objects.	Image processing: erosion and dilatation, connected component labelling technique	Feature extraction (height and width of object’s silhouettes)	Simple threshold-based decision algorithm for fall detection	
Vishwakarma *et al.*[21]	Patient detection (adaptive background subtraction method using Gaussian mixture model)	Feature extraction	Fall detection using aspect ratio and pixel's gradient distribution and applying rule-based decisions	Fall confirmation using the fall angle and applying rule-based decisions	
Cucchiara *et al.*[19]	Extraction of moving objects using background suppression with selective and adaptive update	Tracking algorithm: A probabilistic and appearance-based tracking	Classification as people of tracks that satisfy some geometrical and colour constraints	Posture classifier based on the projection histograms computed over the temporal probabilistic maps obtained by the tracker	Hidden Markov Models formulation is adopted to classify the posture
Fu *et al.*[16]	Extraction of changing pixels (motion events) from the background	A lightweight algorithm computes the instantaneous motion vectors	Fall events are reported using the temporal average of the motion events		
Hazelhoff *et al.*[17]	Object segmentation: (background subtraction and connection of information components)	Object tracking: the tracker can mark objects as non-human, which are identified based on size and absence of both motion and a head region	PCA-based feature extraction: the direction of the principal component and the variance ratio are extracted	Fall detection: using a multi-frame Gaussian classifier	Head tracking using skin-colour model to confirm the fall
Anderson *et al*. [22]	Silhouette extraction from each camera. Then, a 3D representation of the body is constructed	Extraction of centroid, height, major orientation of the body and similarity of the major orientation with the ground plane normal	Human state inferred using fuzzy logic (3 states: upright, on-the-ground and in-between)	Information in sequences of states is reduced by linguistic summarization to produce human readable sentences	Fall detected by a second level of fuzzy logic, taking inputs from a single summary: average state, time duration, speed, oscillation, etc.
Lie *et al.*[23]	Human body identification using frame differencing approach	Image processing: mean filter to make the image more smooth, thresholding to obtain a binary image, connected component labelling	Features extraction and reduction of upper limb activities effect	k-nearest neighbour classifier for human body postures classification	Fall event detection flow: the decision of a fall incident is determined by the event transition and time difference between events
Rimminen *et al.*[28]	Estimate the position of the subject using the near-field image sensor observations	Tracking (Kalman filter) and multi-target tracking (Rao-Blackwellized Monte Carlo data association algorithm)	Features extraction related to the NFI floor	Modelling of the state evolution as a two-state Markov chain (falling, getting up)	Pose estimation using Bayesian filtering. It combines the prior model with information from the features
Tzeng *et al.*[25]	Fall suspection: Thresholding of the floor pressure signal	If the floor preassure exceeds a given threshold: Image capture	Background subtraction through an image thresholding. Objects labelling and expansion (morphological operations)	Image features extraction	Combination of the floor pressure signal and image features to report on a fall
Diraco *et al.*[24]	Camera calibration	Background modelling using Mixture of Gaussians method	Moving regions detection (Bayesian segmentation) and segmented blobs refining (morphological operations and connected components)	Fall suspection: The distance of the centroid from the floor plane is lower than a prefixed value	Fall confirmation if an unchangeable situation persists for at least 4 seconds
Rougier *et al.*[14]	Silhouette detection (foreground segmentation method) and edge points extraction (Canny edge detector)	Silhouette edge points matching through the video sequence	Shape analysis using the mean matching cost and the full Procrustes distance	Fall classification: Gaussian mixture model, based on shape deformation during the fall and the subsequent lack of movement	
Li *et al.*[29]	Locate the position of the sound source	Beamforming to enhance the sound signal using the estimated source position	Mel-frequency cepstral coefficients features are extracted from the sound signal	A nearest neighbour classifier determines if the sound is from a fall	
Mastorakis *et al.*[18]	Feature extraction: width, height and depth of the human posture	Obtaining of the velocities of height and the composite vector of width and depth	When both velocities exceed particular thresholds fall initiation is detected	Inactivity detection: a fall is detected if the height velocity is less than a certain threshold	
Zhang *et al.*[27]	Kinematic Model Based Feature Extraction from Depth Channel	Person tracking by background subtraction	Histogram represented features	Hierarchy Support Vector Machine classification	

The number of subjects involved in the tests is still low if compared to acceleration-based studies (next section). In addition, common to all of these works is the absence of older people during the test period.

Although most of the studies report relatively high accuracies, the experimental findings may not be generalized since there are significant limitations in the test dataset. For example, video-based systems only consider one or two specific sequences in controlled environments [14,15,17,19-24] and other studies with different types of sensors (pressure [25], infrared [18], etc.) only use a few tens of data collected from some young volunteers. Longer real-world tests could probably lead to more realistic results.

Privacy concerns in context aware systems are not minor problems. Methods to protect privacy are dependent on the type of sensor used. In the extreme case of video-based technology, some authors have opted to obscure or distort the person’s appearance in the video to ensure privacy [26]. Although privacy should already be considered in the design stage [27], not all the studies have followed this approach, which is a clear sign that some context aware systems are mainly focused on the technological development rather than on a real-world deployment.

If we focus on the fall detection techniques used in the different studies, a variety of approaches can be found. Table 2 summarizes the contributions of the different authors.

In general, the structure of all methods is very similar. Most of them start with an object detection that can be performed through a background subtraction in the video-based systems [14,15,17,19-23], or from the information provided by the sensors’ observation [28,29]. Some methods also consider a tracking algorithm to filter objects’ position [17,19,27,28]. Then, some features of the detected objects are extracted (Table 1), which should have sufficient discriminative power to identify the fall events. They are used to classify the events as falls or non-falls using a wide range of techniques: Gaussian Mixture Model [14], Rule-based Techniques [21], Multi-frame Gaussian Classifier [17], Bayesian Filtering [28], Nearest-neighbour Rule [23,29], Hidden Markov Models [19], Thresholding Techniques [15,20,25], Fuzzy Logic [22], etc. Some studies confirm that a fall has occurred by performing inactivity detection in the postfall phase [14,18,24].

As a result of this extensive literature search, we found that lots of strategies have been adopted, although currently there is no standardized context-aware technique that was widely accepted by the research community in this field.

### Wearable devices

They can be defined as miniature electronic sensor-based devices that are worn by the bearer under, with or on top of clothing [30]. The vast majority of wearable fall detectors are in the form of accelerometer devices (186 out of 197). Some of them also incorporate other sensors such as gyroscopes to obtain information about the patient’s position. The use of applications based on accelerometers and gyroscopes in gait and balance evaluation, fall risk assessment and mobility monitoring has been actively explored [31]. This trend has increased over the last years due to the availability of cheap embedded sensors included in smartphones. In this paper, we classify the different studies using wearable devices according to whether or not sensors are built into smartphones, 30 and 156 papers respectively. The next two sections provide more details about these subgroups.

#### Accelerometer attached to the body

Acceleration data are collected during falls using independent tri-axial accelerometers attached to different parts of the body. A review of several research studies was conducted. For the purpose of comparison, Table 3 examines the most relevant works. The fields are the same as in section *Context-aware systems*, including a new item with the accelerometer placement on the body. Since Table 3 is only focused on acceleration-based systems, the possible techniques for fall detection are reduced to just two: i) threshold-based methods, TBM, in which a fall is reported when the acceleration peaks, valleys or other shape features reach predefined thresholds; ii) machine learning methods, MLM [32]. The aim is to visualize progress in research over the last years.

**Table 3:** Comparison of acceleration based fall detectors using external accelerometers

**Article**	**Year**	**Basis**	**Detection technique**	**Fall types**	**Subjects**	**Declared perform**	**Position**	**Elders Yes/No**	**Comments**
Lindeman *et al.*[33]	2005	A fall detector placed at head level	TBM considering the spatial direction of the head, the velocity right before the initial contact with the ground and the impact	Falls to the front, side with a 90° turn, back, back with hip flexion.	A young volunteer and an elderly woman (83 years)	High sensitivity and specificity	Ear	Yes	Accelerometers were integrated into a hearing-aid housing, which was fixed behind the ear
				Falls backwards against a wall, while picking up an object and collapse.					
Chen *et al.*[34]	2005	Detect the occurrence of a fall and the location of the victim	TBM considering the impact and the change in orientation	Backward and sideways falls	2 subjects	The acceleration for ADL is much less than the observed from falling	Waist	No	The final orientation of the wearer is considered
Zhang *et al*. [45]	2006	Fall detection using machine learning strategies	MLM. 1) Extraction of temporal and magnitude features from the acceleration signal, 2) One-class Support Vector Machine classifier	Soft fall	12 subjects (8 males, 4 females, ages 10–70)	Accuracy 96,7%	Waist	Yes	To the best of our knowledge, this study is the first in using machine learning techniques
				Hard fall in the ground, stairs and slopes (using a mannequin)					
Bourke *et al.*[35]	2007	Investigation into the ability to discriminate between falls and ADL	TBM using information from the impact	Forward falls, backward falls and lateral falls left and right, performed with legs straight and flexed	10 subjects (ages 21–29)	Trunk	Trunk, thigh	Yes	The trunk appears to be the optimum location for a fall sensor
						SP:100%			
					10 community-dwelling elderly subjects (3 females, 7 males, ages 70–83)	Thigh			
						SP: 83.3%			
Doukas *et al.*[46]	2007	Accelerometers transmit patient movement data wirelessly to the monitoring unit	MLM. The acceleration in the three axis is classified using Support Vector Machine	Not specified	1 subject	SE: 98.2%	Foot	No	If a fall is suspected it also transmits video images to remote monitoring units
						SP: 96.7%			
Kangas *et al.*[36]	2008	Comparison of 3 low-complexity algorithms	TBM considering the start of the fall, the velocity, the impact and the lying posture	Forward, backward, and lateral falls	3 volunteers (1 female, 2 males; ages 38, 42, 48)	Waist	Wrist, head, waist	No	Waist worn accelerometer might be optimal for fall detection considering the fall associated impact and the posture after the fall
						SP: 100%			
						SE: 98%			
Kangas *et al.*[37]	2009	To validate the data collection of a new fall detector prototype	TBM considering two or more of the following phases of a fall event: start of the fall, falling velocity, fall impact, and posture after the fall	Syncope, tripping, sitting on empty air, slipping, lateral fall, rolling out of bed	20 subjects (40–65 years old), 21 voluntary older people (58–98 years old)	SP: 100%	Waist	Yes	Middle-aged persons could be considered to mimic the fall events of older people more adequately than young subjects would
						SE: 97.5%			
Li *et al.*[38]	2009	Fall detection system using both accelerometers and gyroscopes	TBM analyzing the intensity of the activity, the posture and whether the transition to a lying posture was unintentional or not	Forward, backward, sideways and vertical falls. Falling on stairs and fall against walls ending with a sitting position	3 male subjects (age 20)	SP: 92%	Chest, thigh	No	Human activities are divided into static postures and dynamic transitions
						SE: 91%			
Shan *et al.*[42]	2010	Investigation of a pre-impact fall detector	MLM 1) A discriminant analysis is applied to time-domain statistical characteristics to select the features, 2) Support Vector Machine is used for fall recognition	Forward falls, backward falls, lateral falls left and right (subjects were instructed to keep their postures for about 2 seconds after the fall)	5 male subjects (ages 21 – 28)	SP: 100%	Waist	No	Impending falls are detected in their descending phase before the body hits the ground
						SE: 100%			
Bianchi *et al.*[39]	2010	Augmentation of accelerometer-based systems with a barometric pressure sensor	TBM considering the impact, the postural orientation, and the change in altitude associated with a fall	Forward, backward and lateral falls (ending lying, with recovery, with attempt to break the fall)	20 subjects (12 male, 8 female; mean age: 23.7)	SP: 96.5%	Waist	No	A system based on a barometric pressure sensor is compared with an accelerometry-based technique. The acceleration and air pressure data are recorded using a wearable device
						SE: 97.5%			
					5 subjects (2 male, 3 female; mean age: 24)				
				Resting against a wall, then sliding vertically down to the end in the sitting position					
					5 subjects (5 male, mean age: 26.4)				
Bourke *et al.*[40]	2010	It compares novel fall-detection algorithms of varying complexity	TBM considering the fall impact, the velocity and the posture	Forward falls, backward falls, lateral falls left and right all performed with both legs straight and with knees relaxed	10 male subjects (age 24–35)	SP: 100%	Waist	Yes	The algorithms were tested against ADL performed by elderly subjects
					10 older subjects (6 male, 4 female, age 73–90)	SE: 94.6%			
Lai *et al.*[41]	2011	Several acceleration sensors for joint sensing fall events	TBM to differentiate dynamic/static states using the acceleration of the three axis	Forward, backward, rightward or leftward falls	16 subjects	Accuracy 92.92%	Neck, hand, waist, foot	No	After a fall accident occurs, the system determines the level of injury
Bagala *et al.*[9]	2012	Benchmark the performance of published fall-detection methods when they are applied to real-world falls	TBM including, among others, the algorithms published in [35,36]	Real-world falls: indoor/outdoor, forward /backward /sideward, impact against the floor /wall or locker before hitting the floor / sofa or bed/ desk	9 subjects (7 women, 2 men, age: 66.4±6.2)	Average 13 algorithms	Lower back	Yes	Algorithms that were successful at detecting simulated falls did not perform well when attempting to detect real-world falls
						SP: 83.0% ±30.3%			
					15 subjects				
					29 subjects	SE: 57.0%			
					1 subject	±27.3%			
Yuwono *et al.*[43]	2012	Use of a sophisticated fall detection method	MLM. 1) Discrete wavelet transform, 2) Associate a cluster to the input feature vector; fuse cluster information with input, 3) Combined classification (vote majority): Multilayer Perceptron and Augmented Radial Basis Function	Not specified	8 individuals (age 19–28)	SP: 99.6%	Waist	No	Training and clustering are done off-line. Clustering is done using Regrouping particle swarm optimization
						SE: 98.6%			
Kerdegari *et al.*[44]	2012	Investigation of the performance of different classification algorithms	MLM. Input is pre-processed using windowing techniques. Features include acceleration, angular velocity, velocity, position and time domain features: maximum, minimum, mean, range, variance and standard deviation. Several methods are compared.	With flexed knees: forward, backward, sideways falls	50 volunteers (18 male, 32 female, average age 32)	SE: 90.15%	Waist	No	Multilayer Perceptron, Naive Bayes, Decision tree, Support Vector Machine, ZeroR and OneR algorithms are compared.
				Base on wall: backward, sideways falls					
				Backward falls sitting on empty, turning left and right					
									Results show that the Multilayer Perceptron algorithm is the best option
Cheng *et al.*[47]	2013	Daily activity monitoring and fall detection	TBM using a decision tree: 1) A decision tree is applied to the angles of all the body postures to recognize posture transitions, 2) the impact magnitude is thresholded to detect the falls	Four types of falls: from standing to face-up lying, face-down lying, left-side lying, and right-side lying	10 subjects (6 males, 4 females, age 22–26)	SE: 95.33%	Chest	No	Dynamic gait activities are also identified using Hidden Markov Models. Surface electromyography signals are combined with the acceleration signals.
						SP: 97.66%	Thigh		

TBM: Threshold Based Method, MLM: Machine Learning Method.

Most of the existing works use thresholding techniques for automatic fall detection [9,33-41], although the machine learning approach has increased its influence since 2010 [42-44]. The methods applied include Support Vector Machine [42,44-46], Regrouping Particle Swarm Optimization, Gaussian Distribution of Clustered Knowledge [43], Multilayer Perceptron, Naive Bayes, Decision tree [44,47], ZeroR and OneR [44]. The multilayer perceptron seems to be a good supervised option according to Kerdegari et al. [44], although there is no standardized technique that is widely accepted by the scientific community.

The average number of subjects involved in the tests is about 17, which is significantly higher than in context-aware systems (previous section). This indicates a higher reliability of this technology but still insufficient; only 6 of the works involve older people in the ADL study [9,33,35,37,40,45], while the rest use simulated data from young volunteers. As in the previous section, longer real-world tests with target users would be required.

Nearly all the studies concur that the broader categories of typical fall events are forward, backward and sideways, although some of them extend these categories to cover a large number of fall situations [33,38,39]. Regarding the position of the accelerometer, the placement site at the waist seems to be optimal for fall detection. Waist attached accelerometers are located near the body’s centre of gravity, providing reliable information on subject body movements [36].

As in the previous category, the declared performance is very high, but the fact is that there is little use of these devices in daily geriatric practice and no significant industrial deployment of fall detectors due mainly to the significant number of false alarms, resulting in inappropriate alerts [8]. The declared results are valid for laboratory environments with limited data or under restricted conditions, but in real-world scenarios there are lots of uncontrolled factors that lead to a dramatic loss in performance [9]. Therefore, studies should incorporate longer tests and include indicators closer to a real-world usage, for example the number of false alarms per day, which may provide target users with a more realistic idea of the true performance of the system.

#### Smartphone built-in accelerometer

Today’s smartphones come with a rich set of embedded sensors, such as an accelerometer, digital compass, gyroscope, GPS, microphone, and camera [48]. Several researchers are currently taking advantage of this fact to develop smartphone based fall detectors. Table 4 summarizes some relevant works in this field.

**Table 4:** Smartphone based fall detectors

**Article**	**Year**	**Basis**	**Detection technique**	**Fall types**	**Study design**	**Declared perform**	**Position**	**Elders Yes/No**	**Comments**
Sposaro *et al.*[49]	2009	Alert system for fall detection using smart phones	TBM considering the impact, the difference in position before and after the fall and whether the fallen patient is able to regain the upright position	Not included	Not included	Not included	Thigh (pocket)	No	First documented mobile phone-based fall detector
									The existence of a lying period after falling is checked
Dai *et al.*[50]	2010	Mobile phones as a platform for developing fall detection systems	TBM considering the impact, the wearer’s orientation and the common step mechanics during falling	Forward, lateral and backward falls with different speeds (fast and slow) and in different environment (living room, kitchen, etc.)	15 participants from 20 to 30 years old (2 females, 13 males)	Good detection performance	Chest, waist, thigh	No	A detection algorithm with an external accessory is included
Lopes *et al.*[51]	2011	Application to detect and report falls, sending SMS or locating the phone	TBM considering the impact	Fall into bed, forward fall, backward fall, fall in slow motion	Not specified	Not specified	Thigh	No	Five scenarios to validate the detector are presented. Each scenario includes ADL and falls.
Albert *et al.*[54]	2012	Demonstrate techniques to not only reliably detect a fall but also to automatically classify the type	MLMs using a large time-series feature set from the acceleration signal.	Left and right lateral, forward trips, and backward slips	15 subjects (8 females, 7 males, ages 22–50)	Across an average week of everyday movements there are 2–3 non-falls misclassified as falls	Back	No	Five machine learning classifiers are compared: Support vector machines, Sparse multinomial logistic regression, Naïve Bayes, K-nearest neighbours, and Decision trees
Lee *et al.*[52]	2012	Study the sensitivity and specificity of fall detection using mobile phone technology	TBM considering the impact	Forwards, backwards, lateral left and lateral right	18 subjects (12 males, 6 females, ages 29±8.7)	SP: 81%	Waist	No	The motion signals acquired by the phone are compared with those recorded by an independent accelerometer
						SE: 77%			
Fang *et al.*[53]	2012	Fall detection prototype for the Android-based platform	TBM considering the impact and the patient’s orientation	Not specified	4 subjects	SP: 73.78%	Chest, waist thigh	No	Different phone-attached locations are analysed. The chest seems to be the best place.
						SE: 77.22%			
Abbate *et al.*[55]	2012	A system to monitor the movements of patients, recognize a fall, and automatically send a request for help to the caregivers	MLM Eight acceleration properties of fall-like events are classified using multi-layer feed-forward neural network	Forward fall, backward fall, and faint (normal speed and slow motion)	7 volunteers (5 male, 2 female, ages 20–67)	SP: 100%	Waist	No	The proposed approach is compared with the techniques described in [35,36,49,66]
						SE: 100%			

Low-complexity algorithms based on thresholding are used in most of the studies [49-53], and only few go further and adopt machine learning strategies [54,55]. They use Support Vector Machines, Sparse Multinomial Logistic Regression, Naïve Bayes, K-Nearest Neighbors, Decision Trees [54] and Multi-layer Neural Networks [55].

The types of falls considered and the number of subjects involved in the studies are similar to those of the previous section. Regarding the position of the phone, the waist is still the preferred part of the body [52,55], although there is an emerging trend towards the thigh, coinciding with the location of the pocket [49-51,53].

Some of these studies [49] have resulted in real fall detection applications that are available for download in Google Play [56]. This site offers another source of information. Thus, a search has been conducted in this repository including either “fall detector” or “fall detection” as the keywords. As a result, a total of 9 applications were obtained, of which 7 were for seniors. To quote some statistics, 3 of them reported between 1000 and 5000 downloads, while the rest had less than 500. Although these numbers indicate a certain level of interest, they are still far from the number of potential users. Focusing on the app rating, we found that an average of only 6 people have given their opinions on them. This is a symptom that people using these apps do not seem to be enthusiastic about them.

The number of published studies based on smartphones is still low in comparison with the previous categories, and none of them involve older people to evaluate the detector. Therefore, studies still need to incorporate a more exhaustive evaluation. These are signs that we are facing an emerging field.

## Discussion

### Trends, challenges and issues

Based on the extensive literature search, challenges, issues, and trends in fall detection systems have been identified. This section presents the most relevant ones.

### Trends

We start by describing the current and future trends in fall detection systems.

#### Trend 1: Vision and smartphone-based detectors

**Figure 1:**
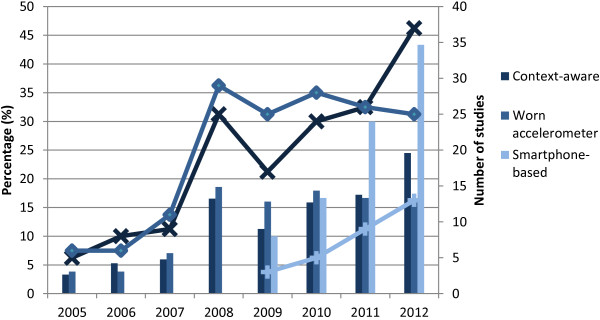
**Estimation of the number of fall detection studies.** We have made a longitudinal study of published papers, classifying the detection techniques into three categories. The line graph (associated with the right axis) represents the estimated absolute number of studies published in the three categories from 2005 to 2012. The bar graph (associated with the left axis) shows the estimated percentage of studies published every year in relation to the total number of existing studies for each category (e.g., 43.3% of the existing smartphone-based studies were published in 2012).

Context-aware techniques appeared over a decade ago and the number of studies is still increasing after a drop some years ago (Figure 1), most of them being video-based systems. It is unfortunate that the algorithmic details of these systems do not seem to converge. Computer Vision approaches are very complex and it is difficult to obtain a system that can work under real life conditions. On the other hand, the use of body-worn accelerometers has stagnated in the last years, but this trend is offset by the increase in the number of smartphone-based studies. In fact, this is still a novel technology: the first study using smartphones appeared in 2009 [49] and since then the research in this field has grown steadily. These are signs that we are facing an emerging trend, which may be explained by the advantages offered by smartphones. As self-contained devices, they present a mature hardware and software environment for developing pervasive fall detection systems [50]. They have built-in communication protocols that allow simple data logging to the device and wireless transmission. Price is also significantly reduced due to high production volume [54,57].

#### Trend 2: Machine learning approach

There are two main approaches to detect falls using acceleration signals: thresholding techniques and machine learning methods. Applications based on the first approach are simple to implement and their computational work is minimal. They are able to detect when a fall occurs. However, the rate of false positives is a significant issue [54]. The machine learning approach is more sophisticated and leads to better detection rates. Nevertheless, there have been documented difficulties with implementing these techniques (for example: requirement of high mathematical skills, use of more computation resources, etc.), although they are currently the prevailing trend, since thresholding methods proved to be ineffective [9]. In addition, no method has been widely accepted and each paper presents a different approach among the variety of machine learning algorithms.

### Challenges

The design of fall detectors faces some major challenges described in this section.

#### Challenge 1: Performance under real-life conditions

Fall detectors need to be as accurate and reliable as possible. A robust fall detection system should exhibit both high sensitivity and specificity. This is sometimes reached in experimental environments, but when applied to a real situation, the detection rate decreases [8]. These devices are designed and tested under controlled conditions, for example they use data from falls and ADL of young people simulated at the discretion of each author due to the lack of a standardized procedure or a public database for comparison. Furthermore, it is worth pointing out that fall detectors are aimed at older people, so they should also be involved in their development. Only few studies incorporate data from older people [9,33,35,37,40,45], although their participation is limited to perform a set of simulated activities of daily living for a few minutes or hours. That is not enough to assess the system performance in a real situation. Users should wear the devices for longer periods (at least months). Some studies have worked in this direction [4,9], resulting in a significant number of false activations, among other concerns.

#### Challenge 2: Usability

Smartphone-based fall detectors are attractive because of the widespread use of phones, even among the older population [58]. However, the majority of the studies referenced in Table 4 placed them in a standardized position. This allowed highly stereotypical measurements that aided accuracy ratings, but made the results less applicable to the way people carry their smartphones every day (for example: in pockets or handbags) [54]. Future smartphone-based detectors should not limit the placement of the device to a single part of the body (waist, wrist, chest, etc.). Smartphones should be used in a normal way, with no restrictions regarding their position or functionalities. This may lead to lower detection rates.

#### Challenge 3: Acceptance

Little is published about the practicality and acceptability of the technology. Elders’ acceptance poses a major problem since they may not be familiar with electronic devices. To overcome this challenge, the way the system operates is essential [59]. The detector should activate and operate automatically, without user intervention. Vision systems, like other non intrusive methods, are very good in this sense. However, some wearable devices like smartphones have other advantages that can help to improve the acceptance of fall detectors. They can operate both indoors and outdoors and integrate not only fall detection but also other healthcare applications in the same device. In this way, the traditional reluctance to carry different devices, each one targeting a specific function, would be overcome. However, the use of smartphones by older people is not without difficulty: these devices, as they were conceived, represent a major usability barrier for them. Proof of this is the absence of rating in the existing fall detection applications, which is a symptom of little real use. In this sense, potential solutions to improve the usability and accessibility of smartphones are needed. Nevertheless, as a result of a study still in progress we have found that fall detectors were highly valued by the elderly, who showed a positive attitude towards smartphone-based solutions after carrying out a practical demonstration of several assistive technologies. This conclusion agrees with the work of Plaza et al. [60], who reviewed mobile applications for older people and found that they are most frequently related to two domains: “Health – wellness – home care” and “Safety – security – mobility”.

### Issues

This section describes the most significant issues which could hinder the system performance.

#### Issue 1: Smartphone limitations

The trend towards smartphone-based detectors poses some problems. Firstly, smartphones are not devices initially intended for fall detection or any other safety critical application [49]. There might be difficulties with real-time operations, the sensing architecture, the stability of the accelerometer’s sampling frequency, the specific features of the operating system, etc. Indeed, the same fall detector might behave slightly differently depending on the smartphone model in which it is installed. This possibility should be taken into account in a real-world scenario. Secondly, smartphones cannot be overloaded with continuous sensing commitments that undermine the performance of the phone, for example by depleting battery power. It is essential to manage the sleep cycle of sensing components in order to trade off the amount of battery consumed [48]. Nonetheless, smartphone’s battery life is always low, which could hinder its acceptance. This is not a minor problem, especially considering that the system is intended for older people with impaired mobility. Thirdly, there is a need for easy-to-use smartphones and here we are in the hands of manufacturers. The potential market of the applications for people with low technical skills will influence the development of adapted devices. Nonetheless, fall detectors are unlikely to reach in the near future the robustness and stability achieved by other assistive technologies such as press-for-help devices.

#### Issue 2: Privacy concerns

Privacy concerns of sensor-based systems, and fall detectors are, have been a hot topic [61]. Of course, not all types of sensors are equally vulnerable: context-aware systems in general, and vision-based systems in particular, are much more prone to privacy concerns than, for example, body-worn acceleration-based devices. In any case, the protection of sensitive context data must be guaranteed [62]. Privacy problems should not prevent the potential benefits of assistive technologies as, at the same time, privacy cannot be sacrificed in order to bring about other benefits [63]. In general, studies on fall detection usually lack strategies to ensure data privacy. This shows that they are still far from a real-life deployment.

#### Issue 3: Comparison among different techniques: public data bases

Comparing different approaches is difficult because each author obtains data in a different way: types of simulated falls and ADL, position of the detector, sampling frequency, temporal length of signal, extracted features, etc. The research focus should be not only on the algorithm to be used but also on the way signals are acquired and treated before feeding a classifier. A public database with accelerometer signals and videos of people falling could help to compare different methods and to improve the reproducibility of the results. Sharing source code of the algorithms would also be a valuable option.

#### Issue 4: Real-life falls

Most studies use data of simulated ADL and falls from young or mature people. Even assuming they were made publicly available for a fair comparison among different methods, it is unclear whether the simulated activities are representative of their real counterparts. Since it is not acceptable to subject older people to simulated falls, the evaluation of the detectors is severely limited. Only few studies present acceleration data from the real-life falls of older people [64,65], but the number of events recorded remain low. In addition, the mechanisms of the falls are not known as they could not be accurately documented [36].

## Conclusion

In conclusion, fall detection is a complex process for which currently there is not a standardized solution. Fall detectors are essential in order to provide a rapid assistance and to prevent fear of falling and their adverse health consequences. This review provides a classification for fall detectors from the analysis of several studies, examines their evolution over time, and ultimately identifies the challenges, issues and trends in fall detection systems.

The number of studies in vision-based systems is still increasing. Besides, there is a new trend towards the integration of fall detectors into smartphones, but their use in real-world scenarios can still be compromised by the factors highlighted in this paper. Both biomedical engineers and clinicians should become aware of the limitations and potential of fall detection systems.

## Abbreviations

ADL: Activities of daily living; SE: Sensitivity; SP: Specificity; TBM: Threshold-based method; MLM: Machine Learning Method.

## Competing interests

The authors declare that they have no competing interests.

## Authors’ contributions

RI and CM carried out the research and drafted the manuscript. IP participated in its design, performed the statistical analysis and revised it critically for important intellectual content. All authors read and approved the final manuscript.
